# Changes in the Number of Gastrointestinal Cancers and Stage at Diagnosis with COVID-19 Pandemic in Japan: A Multicenter Cohort Study

**DOI:** 10.3390/cancers15174410

**Published:** 2023-09-04

**Authors:** Kento Kuzuu, Noboru Misawa, Keiichi Ashikari, Shigeki Tamura, Shingo Kato, Kunihiro Hosono, Masato Yoneda, Takashi Nonaka, Shozo Matsushima, Tatsuji Komatsu, Atsushi Nakajima, Takuma Higurashi

**Affiliations:** 1 Department of Gastroenterology and Hepatology, Yokohama City University School of Medicine, 3-9 Fukuura, Kanazawa-ku, Yokohama 236-0004, Japan; kentokuzuu@yahoo.co.jp (K.K.); nobomisa@yokohama-cu.ac.jp (N.M.); ashikari@yokohama-cu.ac.jp (K.A.); shigeki4321@gmail.com (S.T.); shin800m@yokohama-cu.ac.jp (S.K.); hiro1017@yokohama-cu.ac.jp (K.H.); yoneda@yokohama-cu.ac.jp (M.Y.); nrk20799@nifty.com (A.N.); 2 National Hospital Organization Yokohama Medical Center, 3-60-2 Harazyuku, Totuka-ku, Yokohama 245-8575, Japan; nonaka_taka@yahoo.co.jp (T.N.); matsushima.shozo.bu@mail.hosp.go.jp (S.M.); komatsu.tastuji.je@outlook.jp (T.K.)

**Keywords:** COVID-19, new normal, gastrointestinal cancer, cancer, stage, detection process, Japan

## Abstract

**Simple Summary:**

The number of gastrointestinal cancer patients declined during the COVID-19 pandemic. In Japan, the number of early-stage gastric and colorectal cancer patients decreased significantly. However, how gastrointestinal cancers have changed with the shift to the “new normal” remains unknown. Herein, we compared the number of patients, stage at diagnosis, and detection process of the different gastrointestinal cancers in Japan across three periods: pre-COVID-19, Midst of COVID-19 pandemic, and during the transitional period to the “new normal”. The number of colorectal cancer patients decreased in the early phase of the COVID-19 pandemic. It then increased with the shift to the “new normal”, no longer differing from pre-COVID-19 levels. However, gastric cancer patients remained significantly fewer in the “new normal” period than in the pre-COVID-19 period, especially Stage I patients and patients detected through screening. Thus, people with undetected gastric cancer may not be receiving optimal screening, implying a negative prognostication for the near future.

**Abstract:**

This retrospective cohort study compared the number of newly diagnosed patients, stage at diagnosis, and detection process of gastrointestinal cancers based on hospital-based cancer registry data at two tertiary Japanese hospitals. The pre-COVID-19 period was from January 2017 to February 2020, with phase 1 (midst of COVID-19 pandemic) from March to December 2020 and phase 2 (the transition period to the “new normal”) from January to December 2021. Each month, the number of patients diagnosed with esophageal, gastric, colorectal, pancreatic, liver, and biliary tract cancers were aggregated, classified by stage and detection process, and compared, including a total of 6453 patients. The number of colorectal Stage 0-II patients decreased significantly in phase 1 and increased in phase 2. The total number of colorectal cancer patients returned to pre-COVID-19 levels (mean monthly patients [SD]: 41.61 [6.81] vs. 36.00 [6.72] vs. 46.00 [11.32]). The number of patients with gastric cancer Stage I significantly decreased in phase 2 following phase 1. The number of gastric cancer patients decreased significantly from pre-COVID-19 levels (30.63 [6.62] vs. 22.40 [5.85] vs. 24.50 [4.15]). During phase 2, the number of patients diagnosed after screening with colorectal cancer increased significantly, whereas that with gastric cancer remained considerably lower. The number of Stage III colorectal and gastric cancer patients increased significantly from the pre-COVID-19 levels. Thus, gastric cancer may not be optimally screened during phases 1 and 2. There was a significant increase in patients with Stage III colorectal and gastric cancers from the pre-COVID-19 period; hence, the stage at diagnosis may have progressed.

## 1. Introduction

The first case of COVID-19 was confirmed in Wuhan, China, in December 2019, after which the disease spread worldwide, leading to a pandemic. At least one-third of people infected with SARS-CoV-2 remain asymptomatic, but they can continue to spread the virus through close personal contact [1]. Therefore, many governments have implemented lockdowns or declared states of emergency to prevent the spread of SARS-CoV-2. In Japan specifically, the infection began spreading in March 2020, leading to the declaration of a state of emergency from 7 April to 25 May 2020, and a call for self-quarantine.

Consequently, the number of hospital visits and medical examinations decreased during this period [2,3,4,5,6,7,8,9]. The effects were manifold. For example, the number of newly diagnosed heart failure and stroke patients declined, and the proportion of severely ill patients increased, which could be related to decreased consultations for patients with relatively minor illnesses and symptoms [10,11,12]. Conversely, the number of newly diagnosed diabetes patients increased, as did the number of diabetic ketoacidosis patients, which could be related to delays in hospital visits, lifestyle changes, and stress [13]. Additionally, there were many reports that the number of newly diagnosed cancer patients decreased during the lockdown [14,15,16,17].

In 2020, SARS-CoV-2 was a poorly understood virus with a high fatality rate [18,19]. No vaccines were available, and self-quarantine was the only known infection control measure [20,21,22,23]. Thus, in our previous study, we extended the observation period until the end of 2020 and investigated the impact of the COVID-19 pandemic on gastrointestinal cancers in Japan. We reported that in 2020, the number of patients with early gastric and colorectal cancers decreased significantly and colorectal cancer staging may have progressed. We expected to observe further negative effects in the future [24].

Similar results were subsequently reported from several countries. An Italian study reported that in 2020, the proportion of colorectal cancer patients decreased by 29%, and the proportion of patients with early-stage colorectal cancer was particularly small. They suggest that this was due to a lack of medical resources [25]. A Dutch study reported a decrease in esophageal cancer patients in 2020 and an increase in the proportion of patients with incurable esophageal and stomach cancer. They suggest that this was owing to patients with mild symptoms refraining from medical visits [26]. An English study reported a 37% decrease in hepatocellular carcinoma patients in 2020. They suggest that this was due to crowding in primary care services [27].

In 2021, sooner than expected, with the development of a vaccine, evolution of viral variants, and a decline in mortality rate, the infection control measures gradually shifted from self-quarantine to the “new normal” [28,29,30]. Social activities increased and travel was resumed [31,32]. The number of COVID-19 patients increased from 230,000 in 2020 to 1,500,000 in 2021, indicating that opportunities for contact increased [33]. Accordingly, the number of people receiving medical checkups reportedly recovered to the same level as that during the pre-COVID-19 period [6,7,8,9]. However, it is not yet known whether the number of gastrointestinal cancer patients has recovered as we shift to the “new normal”, and whether the progression of gastrointestinal cancer has been suppressed in Japan.

In this study, we compared the number of gastrointestinal cancer patients, stages, and detection processes in 2021, the year of transition to the “new normal”, with that in 2020 and before COVID-19, using the same cancer registry database [24]. To further understand the effect of the COVID-19 pandemic on the diagnosis of gastrointestinal cancers, we also thoroughly investigated the cancer detection process. This is the first study from Japan that studies the effects of the COVID-19 pandemic on gastrointestinal cancers according to the stage and detection process of cancers until 2021.

## 2. Methods

### 2.1. Study Design and Setting

This retrospective study evaluated patients newly diagnosed with gastrointestinal cancer at Yokohama City University Hospital and the National Hospital Organization Yokohama Medical Center between January 2017 and December 2021. These are regional core and tertiary emergency hospitals. Despite caring for patients with severe COVID-19, neither hospital had a cluster outbreak. Therefore, these two hospitals did not restrict consultation, examination, surgery, or chemotherapy. However, the Japan Gastroenterological Endoscopy Society recommended that gastroscopies and colonoscopies be postponed in asymptomatic patients until after the state of emergency (April and May 2020) owing to the high risk of infection these procedures carry, which these hospitals complied with. 

This report followed the Strengthening the Reporting of Observational Studies in Epidemiology (STROBE) guidelines. This retrospective study protocol was approved by the ethics committees of Yokohama City University Hospital (9 March 2023) and the National Hospital Organization Yokohama Medical Center (15 February 2023). The patients were allowed to opt out of the study via the hospital website.

### 2.2. Patients and Cancer Registry

Cancer patients were identified from hospital-based cancer registries that are part of the National Cancer Registry [34]. Suspected cancer patients were identified based on the name of the disease, pathological findings, history of chemotherapy, surgery, radiation therapy, palliative care, and referral to cancer centers, and aggregated into a single list. The cancer registries are populated by registrants certified by the National Cancer Center, Japan.

Gastrointestinal cancers were defined according to the International Statistical Classification of Diseases and Related Health Problems, Tenth Revision (ICD-10) codes: C15 indicates esophageal cancer, C16 indicates gastric cancer, C18 to C20 indicate colorectal cancer, C22 indicates hepatocellular carcinoma, C23 to C24 indicate biliary tract cancer, and C25 indicates pancreatic cancer. Neuroendocrine tumors or lymphomas were excluded. Intrahepatic cholangiocarcinoma was excluded because it is clinically and pathologically different from hepatocellular carcinoma (C22). Patients were considered eligible if they were diagnosed or started their first treatment including palliative care at the study hospitals and were considered ineligible if they were diagnosed and started their first treatment at other hospitals.

### 2.3. Data Extraction

Data on patient sex, age, date of diagnosis, detection process, clinical stage (based on the Union for International Cancer Control staging system, eighth edition), and first treatment were extracted from hospital-based cancer registries. Patients without clearly identified cancer stage on the registries were entered based on the information obtained from their medical records. The date of diagnosis was defined as the date of the first clinical examination performed to diagnose cancer (not the date of the pathological diagnosis).

### 2.4. Outcomes

The period from January 2017 to February 2020 was defined as the pre-COVID-19 period. The period from March to December 2020, when self-quarantine was the predominant pattern of infection control, was defined as COVID-19 pandemic phase 1. Further, the period from January to December 2021, when vaccination was initiated and social activities gradually resumed, was defined as COVID-19 pandemic phase 2. The number of patients newly diagnosed with cancer were aggregated for each month, classified according to stage, and compared. 

The detection process was categorized as either a medical checkup, screening, or symptomatic cases. A medical checkup includes examinations conducted by a company, school, municipality, or at the patient’s own expense. Screening includes examination for patients who are at risk of cancers and who are being followed up for other diseases.

### 2.5. Statistical Analysis

Patient sex was analyzed using χ^2^ test and patient age was analyzed using Analysis of Variance (ANOVA). The number of gastroenterology patients and endoscopies were aggregated by month and analyzed using ANOVA and Steel–Dwass tests. The number of patients by cancer type, stages, and detection process were aggregated by month and analyzed using ANOVA and Tukey–Kramer tests. Differences were considered statistically significant at *p* < 0.05. Statistical analyses were performed using the JMP Pro 17 software (SAS Institute, Cary, NC, USA).

## 3. Results

### 3.1. Patient Characteristics

The study evaluated 6453 patients, including 4218 pre-COVID-19, 949 in phase 1, and 1286 in phase 2 (Table 1). The proportion of male patients (2825 [67.0%] vs. 607 [64.0%] vs. 826 [64.2%]; *p* = 0.069) and the mean age (71.3 ± 10.9 vs. 71.8 ± 10.7 vs. 71.9 ± 11.8; *p* = 0.145) did not differ significantly according to period or cancer type during each period.

The number of initial gastroenterology visits was 22,696; the monthly average number of initial visits was 394 ± 33.8 vs. 332 ± 88.3 vs. 365 ± 34.9 (Table 2). Compared with that in the pre-COVID-19 period, there was a significant decrease in Phase 1 (*p* = 0.046). However, the difference was not significant in Phase 2 (*p* = 0.062). The number of gastroenterology follow-up visits, gastroscopies, and colonoscopies also followed a similar trend, with a decrease in phase 1 and an increase in phase 2, none of which was significant.

### 3.2. Colorectal Cancer

The number of patients with colorectal cancer was 2493; the average number of patients per month was 41.61 ± 6.81 vs. 36.00 ± 6.72 vs. 46.00 ± 11.3 (Table 3). The number of patients decreased in phase 1 and significantly increased in phase 2 compared with that in phase 1 (*p* = 0.012). It was higher than the pre-COVID-19 levels. The number of patients per month with Stage 0 (10.58 ± 3.36 vs. 7.10 ± 4.09 vs. 11 ± 3.91), Stage I (10.16 ± 3.14 vs. 6.70 ± 2.91 vs. 9.42 ± 2.50), and Stage II (7.42 ± 3.06 vs. 4.80 ± 1.75 vs. 6.00 ± 2.86) decreased significantly in phase 1 but increased in phase 2 until there was no longer a significant difference compared with the pre-COVID-19 levels.

The number of patients per month with Stage III (7.18 ± 2.85 vs. 12.10 ± 2.42 vs. 11.9 ± 3.64) increased significantly in phase 1 (*p* < 0.001) and continued to increase significantly in phase 2 (*p* < 0.001) compared with the pre-COVID-19 levels.

Although the number of patients per month detected via symptoms (20.95 ± 5.05 vs. 20.50 ± 3.44 vs. 19.58 ± 7.86) remained unchanged over the entire study period, that detected by medical checkup (5.45 ± 2.36 vs. 4.10 ± 1.91 vs. 8.75 ± 3.19) and screening (13.84 ± 4.06 vs. 10.10 ± 2.38 vs. 16.50 ± 4.01) decreased in phase 1 and increased significantly in phase 2 (*p* < 0.001).

### 3.3. Gastric Cancer

The number of patients with gastric cancer was 1682; the average number of patients per month was 30.6 ± 6.62 vs. 22.4 ± 5.85 vs. 24.5 ± 4.15 (Table 4). The number of patients detected in phase 2 was higher than that in phase 1. However, it was still significantly decreased compared with the pre-COVID-19 (*p* = 0.001) levels. The number of patients per month with Stage I (21.6 ± 5.66 vs. 13.9 ± 5.99 vs. 15.75 ± 3.84) increased in Phase 2 compared with that in Phase 1. It was still significantly decreased compared with the pre-COVID-19 levels (*p* = 0.006). In contrast, the number of patients per month with Stage III (1.94 ± 1.37 vs. 1.40 ± 1.07 vs. 3.25 ± 2.05) significantly increased in phase 2 from that in the pre-COVID-19 period (*p* = 0.03).

Similar to colorectal cancer, the number of gastric cancer patients per month detected via symptoms (10.68 ± 3.65 vs. 10.20 ± 2.82 vs. 9.25 ± 2.93) remained unchanged over the entire period, while the number of those detected by medical checkup (5.58 ± 2.20 vs. 3.70 ± 1.34 vs. 6.42 ± 3.06) decreased in phase 1 and increased significantly in phase 2 (*p* = 0.02). In contrast, the number of patients per month detected by screening (12.97 ± 3.61 vs. 8.00 ± 2.54 vs. 8.50 ± 2.39) decreased significantly in phase 1 and remained almost unchanged in phase 2 (*p* < 0.001).

### 3.4. Pancreatic Cancer

The number of patients with pancreatic cancer was 844; the average number of patients per month was 14.0 ± 3.37 vs. 14.1 ± 3.32 vs. 14.3 ± 4.18 (Table 5). The number of patients detected during phase 2 did not increase (*p* = 0.98) and remained stable throughout the entire study period. No significant differences were observed according to stage. The number of patients detected by screening (4.05 ± 1.77 vs. 3.70 ± 1.49 vs. 5.33 ± 2.93) increased in phase 2; however, the change was not significant (*p* = 0.15).

### 3.5. Esophageal Cancer

The number of patients with esophageal cancer was 514; the average number of patients per month was 8.82 ± 2.75 vs. 8.70 ± 2.71 vs. 7.67 ± 3.65 (Table 6). The number of patients per month decreased slightly in phase 2 compared to the pre-COVID-19 levels (*p* = 0.47). Only the number of patients with Stage II (0.97 ± 1.03 vs. 0.80 ± 0.79 vs. 1.33 ± 1.15) increased from the pre-COVID-19 levels. The number of patients per month detected by screening (3.39 ± 1.57 vs. 3.00 ± 1.33 vs. 2.42 ± 1.62) decreased in phase 2 compared with that in the pre-COVID-19 period, while those detected by medical checkups (0.97 ± 0.94 vs. 1.10 ± 0.88 vs. 1.67 ± 1.30) increased slightly in Phase 2. However, the changes were not significant.

### 3.6. Hepatocellular Carcinoma

The total number of patients with hepatocellular carcinoma was 493; the average number of patients per month was 8.89 ± 3.17 vs. 7.50 ± 2.27 vs. 6.67 ± 2.61 (Table 7). There was no significant difference; however, the number of patients decreased in phase 2 (*p* = 0.066). The number of patients with all stages decreased from the pre-COVID-19 level to that in in phase 2; however, the changes according to stages were not significant. The number of patients detected by screening (6.55 ± 2.84 vs. 4.60 ± 1.65 vs. 4.75 ± 2.14) in phase 2 decreased compared with the pre-COVID-19 levels (*p* = 0.09). However, the change was not significant.

### 3.7. Biliary Tract Cancer

The total number of patients with biliary tract cancer was 427; the average number of patients per month was 7.05 ± 3.06 vs. 6.20 ± 2.25 vs. 8.08 ± 3.73 (Table 8). The number of patients increased in phase 2 to a non-significant degree from the pre-COVID-19 levels (*p* = 0.58). Numbers according to stages did not differ significantly compared with the pre-COVID-19 levels. However, the number of patients with Stage III (1.55 ± 1.37 vs. 1.20 ± 1.03 vs. 2.58 ± 1.24) increased significantly from phase 1 to phase 2 (*p* = 0.04). There was no significant change according to the detection process. However, the number of patients detected by symptoms (4.42 ± 2.10 vs. 4.40 ± 1.84 vs. 5.33 ± 2.87) increased the most compared with the pre-COVID-19 levels (*p* = 0.44).

## 4. Discussion

This is the first study from Japan to investigate the effects of COVID-19 on gastrointestinal cancers by the stage and detection process of cancers from before the pandemic until 2021. In a previous study during phase 1 of the COVID-19 pandemic, the number of patients with Stage I gastric cancer and Stage 0-II colorectal cancer significantly decreased. This was suspected to be caused by a decrease in the number of cancers detected by medical checkup [24]. At that time, the COVID-19 pandemic had been prolonged, and the impact of self-quarantine was expected to be even more pronounced. However, the infection control measures gradually shifted from self-quarantine to the “new normal” earlier than expected, and socioeconomic activities were resumed. Accordingly, the number of gastroenterology initial and follow-up visits decreased in phase 1, but increased in phase 2 and there was no longer a significant difference compared with the pre-COVID-19 period. The number of gastroscopies and colonoscopies also increased in phase 2 to the same level as that during the pre-COVID-19 period. 

The average number of colorectal cancer patients per month in phase 2 was higher than that during the pre-COVID-19 period. The number of Stage 0 and I cases, which decreased significantly in phase 1, returned to the same level as that of the pre-COVID-19 period. This could be due to an increase in the number of patients detected by screening as well as by medical checkups. The screening method for colorectal cancer is the fecal occult blood test, which is easy to perform. This could have contributed to this increase. The number of Stage III cases increased significantly in phase 2. It is possible that the overall stage of presenting cancer may have progressed. Several international studies have suggested the progression of colorectal cancer [9,35,36]. Colorectal cancer could progress if the time from the positive fecal occult blood test to colonoscopy exceeds 10 months [37,38,39,40] or if the time from diagnosis to surgery exceeds 3 months [41,42]. Hence, the rate of progression may not be slow after the development of advanced cancer, even though adenomas might take years to become cancerous [43]. Since the number of cases detected by screening and the total number of patients has increased, it may be important to shorten the time from fecal occult blood positivity to colonoscopy to control stage progression.

Several studies have reported a decrease in gastric cancer cases in 2020. A decrease in Stage I cases has also been observed in other regions [44,45]. In this study, the number of colorectal cancer patients significantly increased in phase 2 compared to that in phase 1. However, the average number of gastric cancer patients per month remained significantly lower than the pre-COVID-19 level, even in phase 2. The number of gastric cancer cases detected through medical checkups has increased from the pre-COVID-19 level. However, unlike colorectal cancer, the number of patients detected by screening remains low. The continuing decline in the patients could be attributed to fewer patients being detected by screening. In other words, in Japan, there were no restrictions on medical visits or instructions to postpone examinations, but it is possible that appropriate screening was not performed for gastric cancer. Unlike fecal occult blood tests for colorectal cancer, there is no easy screening method for gastric cancer, which could lead to a lower number of detected cases. In addition, the trend of decreasing patient numbers could also be influenced by the decrease in *H. pylori* infection rate. In contrast, the number of stage III gastric cancer patients during phase 2 has increased significantly compared to the pre-COVID-19 levels. A few studies have suggested the progression of gastric cancer as compared with those reporting the progression of colorectal cancer [46,47]. However, as gastric cancer has a poorer 5-year survival rate than colorectal cancer, this progression could be expected [34]. Only gastric cancer cases decreased significantly, even in Phase 2, suggesting the possibility of future negative effects. Since the number of cases detected by screening is still low, physicians should actively recommend gastroscopy for patients at high risk of gastric cancer.

The number of patients with pancreatic cancer and their stages did not change significantly in phase 2. This could be because pancreatic cancer is often detected by symptoms. Pancreatic cancer also has several known risk factors, including diabetes. Screening methods for pancreatic cancer, including echography and computer tomography, are also less painful for patients [48]. Therefore, screening could have been performed without delay for patients as needed. Since pancreatic cancer has been less affected by the pandemic, we should continue to focus on detecting pancreatic cancer during its early stages.

The number of patients with esophageal cancer decreased slightly in phase 2, with no significant change in stages. The number of cases detected by medical checkups increased and that detected by screening decreased. As few cases are detected by medical checkups, the decrease in the number detected by screening could have led directly to a decrease in the number of cases. The only approach to detect early-stage esophageal cancer is gastroscopy, similar to gastric cancer. Hence, sufficient screening may not have been performed. It is important to actively recommend gastroscopy to patients at high risk of esophageal cancer.

There was no significant change in the number of hepatocellular carcinoma cases. However, the average number of patients per month decreased further in phase 2. All stages decreased from those during the pre-COVID-19 period, with no stage progression. British and Italian studies have reported a similar trend; this could be a characteristic feature of hepatocellular carcinoma [27,49]. The majority of patients with hepatocellular carcinoma were originally diagnosed via screening. However, in phase 2, the number of cases detected by screening was fewer than that during the pre-COVID-19 period, which could have contributed to the decrease in the number of patients. Patients with viral hepatitis, alcoholic cirrhosis, or fatty liver disease, which are risk factors for hepatocellular carcinoma, often do not visit hospitals as they are asymptomatic. Therefore, they may have lost the opportunity for regular follow-ups and new hospital visits because of the COVID-19 pandemic. To return the number of patients with hepatocellular carcinoma to pre-COVID-19 levels, we might have to focus on identifying patients at a high risk of hepatocellular carcinoma.

There was no significant change in biliary tract cancer cases. However, the average number of patients per month increased in phase 2 from the pre-COVID-19 period, which could be a reaction to the decrease in phase 1. While not significantly different, the number of patients with Stages 0-I decreased and those with Stages II-IV increased from that during the pre-COVID-19 period. This could indicate the progression of stages. Biliary tract cancer has the second poorest prognosis after pancreatic cancer, and it progresses rapidly [25]. However, its risk factors are not well established [50] and its screening is more difficult than that for pancreatic cancer. Therefore, a larger proportion of patients are detected by symptoms than those with pancreatic cancer. It is important to shorten the time between the onset of symptoms such as jaundice and hospital visits. Hence, it is important to disseminate the awareness of biliary tract cancer widely and to encourage patients to visit a hospital as early as possible.

This study had some limitations. First, we only collected data until the end of 2021. This may not be sufficient to fully clarify the effects of the COVID-19 pandemic, as we shifted even more into the “new normal” in 2022 and 2023. Second, we collected data from two Japanese hospitals, which might have limited the power of the analyses. We did not consider interhospital differences. Thus, including a larger sample of patients from more institutions may provide more representative results. Third, as this retrospective study only counted the number of patients diagnosed per month, factors besides the COVID-19 pandemic were not considered. However, in this specific area, there were no additional disasters or significant changes in population numbers, new hospitals, or number of staff members. Fourth, we only evaluated patients with universal health insurance in Japan. We cannot comment on whether differences may emerge in different regions or socioeconomic classes.

## 5. Conclusions

The effects of the COVID-19 pandemic on gastrointestinal cancers and the correlating medical services were expected to be long-lasting. However, by 2021, the effects were already diminishing, and the number of colorectal cancer cases have increased from the pre-COVID-19 levels. The number of gastric cancer cases remains significantly lower, especially those detected by screening. Thus, it is important to aggressively recommend gastroscopy to patients at high risk of gastric cancer. There was also a significant increase in the number of patients with Stage III gastric and colorectal cancers from the pre-COVID-19 period, suggesting the possibility that the stages may have progressed. By evaluating the effects of the COVID-19 pandemic in this study, we determined the trends and characteristics of each type of gastrointestinal cancer, which could be useful for future cancer control measures.

## Figures and Tables

**Table 1:** Patient characteristics.

	Pre-COVID-19 ^a^	Phase 1 ^b^	Phase 2 ^c^	*p*-Value ^d^
Gastrointestinal cancers				
Total, *n*	4218	949	1286	
Age, mean ± SD	71.3 ± 10.9	71.8 ± 10.7	71.9 ± 11.8	0.15
Men, *n*(%)	2825(67.0%)	607(64.0%)	826(64.2%)	0.07
Women, *n*(%)	1393(33.0%)	342(36.0%)	460(35.8%)	
Colorectal cancer				
Total, *n*	1581	360	552	
Age, mean ± SD	70.4 ± 11.6	70.8 ± 11.6	70.8 ± 12.3	0.74
Men, *n*(%)	978(61.9%)	208(57.8%)	339(61.4%)	0.36
Women, *n*(%)	603(38.1%)	152(42.2%)	213(38.6%)	
Gastric cancer				
Total, *n*	1164	224	294	
Age, mean ± SD	72.5 ± 10.0	73.5 ± 9.3	73.7 ± 11.4	0.10
Men, *n*(%)	836(71.8%)	160(71.4%)	191(65.0%)	0.07
Women, *n*(%)	328(28.2%)	64(28.6%)	103(35.0%)	
Pancreatic cancer				
Total, *n*	532	141	171	
Age, mean ± SD	69.4 ± 11.5	71.0 ± 10.8	70.9 ± 12.6	0.17
Men, *n*(%)	311(58.5%)	73(51.8%)	100(58.5%)	0.34
Women, *n*(%)	221(41.5%)	68(48.2%)	71(41.5%)	
Esophageal cancer				
Total, *n*	335	87	92	
Age, mean ± SD	71.6 ± 9.1	71.8 ± 10.0	71.3 ± 10.7	0.94
Men, *n*(%)	275(82.1%)	68(78.2%)	78(84.8%)	0.51
Women, *n*(%)	60(17.9%)	19(21.8%)	14(15.2%)	
Hepatocellular carcinoma				
Total, *n*	338	75	80	
Age, mean ± SD	73.5 ± 10.8	72.5 ± 10.4	73.3 ± 11.3	0.78
Men, *n*(%)	244(72.2%)	57(76.0%)	63(78.8%)	0.44
Women, *n*(%)	94(27.8%)	18(24.0%)	17(21.2%)	
Biliary tract cancer				
Total, *n*	268	62	97	
Age, mean ± SD	72.2 ± 10.2	72.8 ± 10.6	74.1 ± 8.8	0.26
Men, *n*(%)	181(67.5%)	41(66.1%)	55(56.7%)	0.16
Women, *n*(%)	87(32.5%)	21(33.9%)	42(43.3%)	

^a^ Pre-COVID-19 period was defined as January 2017 to February 2020. ^b^ Phase 1 (midst of COVID-19 pandemic) was defined as March–December 2020. ^c^ Phase 2 (transition period to “new normal”) was defined as January–December 2021. ^d^ *p*-values were calculated using χ^2^ test and analysis of variance (*p* < 0.05).

**Table 2:** Comparison of the number of gastroenterology patients and endoscopies.

Background	Number of Patients per Month, Mean (SD)			*p*-Value ^a^		
	Pre-COVID-19	Phase 1	Phase 2	Pre-COVID-19 vs. Phase 1	Pre-COVID-19 vs. Phase 2	Phase 1 vs. Phase 2
First visit	394(33.8)	332(88.3)	365(34.9)	0.046 *	0.06	0.60
Follow-up visit	4458(279)	4128(564)	4291(382)	0.31	0.37	0.97
Gastroscopy	736(84.2)	693(125)	755(69.2)	0.54	0.85	0.46
Colonoscopy	441(45.3)	399(82.9)	436(25.6)	0.42	0.96	0.79

^a^ *p*-values were calculated using the Steel–Dwass test (* *p* < 0.05).

**Table 3:** The average number of colorectal cancer patients per month per stage, the detection process, and comparison between each period.

	Number of Patients per Month, Mean (SD)			*p*-Value ^a^		
	Pre-COVID-19	Phase 1	Phase 2	Pre-COVID-19 vs. Phase 1	Pre-COVID-19 vs. Phase 2	Phase 1 vs. Phase 2
Colorectal cancer						
Total	41.61(6.81)	36.00(6.72)	46.00(11.32)	0.12	0.22	0.01 *
Stage 0	10.58(3.36)	7.10(4.09)	11.00(3.91)	0.02 *	0.93	0.04 *
Stage I	10.16(3.14)	6.70(2.91)	9.42(2.50)	0.01 *	0.74	0.09
Stage II	7.42(3.05)	4.80(1.75)	6.00(2.86)	0.03 *	0.3	0.59
Stage III	7.18(2.85)	12.10(2.42)	11.92(3.96)	<0.001 *	<0.001 *	0.99
Stage IV	6.26(3.13)	5.30(2.83)	7.67(2.99)	0.65	0.36	0.18
Medical Checkup cases ^b^	5.45(2.36)	4.10(1.91)	8.75(3.19)	0.29	<0.001 *	<0.001 *
Screening cases ^b^	13.84(4.06)	10.10(2.38)	16.50(4.01)	0.02 *	0.10	<0.001 *
Symptomatic cases ^b^	20.95(5.05)	20.50(3.44)	19.58(7.86)	0.92	0.74	0.97

^a^ *p*-values were calculated using the Tukey–Kramer test (* *p* < 0.05). ^b^ The detection process was categorized into medical checkups (conducted by the company, school, municipality, or at the patient’s own expense), screening (for patients who are at risk for cancers and who are being followed up for other diseases), and symptomatic cases.

**Table 4:** The average number of gastric cancer patients per month per stage, the detection process, and comparison between each period.

	Number of Patients per Month, Mean (SD)			*p*-Value ^a^		
	Pre-COVID-19	Phase 1	Phase 2	Pre-COVID-19 vs. Phase 1	Pre-COVID-19 vs. Phase 2	Phase 1 vs. Phase 2
Gastric cancer						
Total	30.63(6.62)	22.40(5.85)	24.50(4.15)	0.001 *	0.01 *	0.70
Stage I	21.55(5.66)	13.90(5.99)	15.75(3.84)	<0.001 *	0.01 *	0.71
Stage II	2.71(1.59)	2.20(1.4)	2.17(2.08)	0.67	0.59	1.00
Stage III	1.97(1.37)	1.40(1.07)	3.25(2.05)	0.53	0.03 *	0.01 *
Stage IV	4.39(2.02)	4.90(3.51)	3.33(1.5)	0.80	0.33	0.24
Medical Checkup cases ^b^	5.58(2.20)	3.70(1.34)	6.42(3.06)	0.06	0.51	0.02 *
Screening cases ^b^	12.97(3.61)	8.00(2.54)	8.50(2.39)	<0.001 *	<0.001 *	0.93
Symptomatic cases ^b^	10.68(3.65)	10.20(2.82)	9.25(2.93)	0.92	0.42	0.79

^a^ *p*-values were calculated using the Tukey–Kramer test (* *p* < 0.05). ^b^ The detection process was categorized into medical checkups (conducted by the company, school, municipality, or at the patient’s own expense), screening (for patients who are at risk for cancers and who are being followed up for other diseases), and symptomatic cases.

**Table 5:** The average number of pancreatic cancer patients per month per stage, the detection process, and comparison between each period.

	Number of Patients per Month, Mean (SD)			*p*-Value ^a^		
	Pre-COVID-19	Phase 1	Phase 2	Pre-COVID-19 vs. Phase 1	Pre-COVID-19 vs. Phase 2	Phase 1 vs. Phase 2
Pancreatic cancer						
Total	14.00(3.37)	14.10(3.31)	14.25(4.18)	1.00	0.98	0.99
Stage 0	0.50(0.60)	0.80(0.79)	0.25(0.45)	0.36	0.44	0.10
Stage I	3.34(2.45)	2.50(1.58)	3.83(1.70)	0.53	0.78	0.34
Stage II	2.45(1.54)	2.40(1.71)	1.67(1.50)	1.00	0.29	0.52
Stage III	2.08(1.58)	2.30(1.64)	2.58(2.11)	0.93	0.65	0.92
Stage IV	5.63(2.31)	6.10(2.51)	5.92(1.38)	0.82	0.92	0.98
Medical Checkup cases ^b^	1.00(0.99)	0.80(0.63)	1.25(0.97)	0.82	0.70	0.50
Screening cases ^b^	4.05(1.77)	3.70(1.49)	5.33(2.93)	0.87	0.14	0.15
Symptomatic cases ^b^	8.42(2.97)	9.00(2.49)	7.33(2.71)	0.84	0.49	0.37

^a^ *p*-values were calculated using the Tukey–Kramer test (*p* < 0.05). ^b^ The detection process was categorized into medical checkups (conducted by the company, school, municipality, or at the patient’s own expense), screening (for patients who are at risk for cancers and who are being followed up for other diseases), and symptomatic cases.

**Table 6:** The average number of esophageal cancer patients per month per stage, the detection process, and comparison between each period.

	Number of Patients per Month, Mean (SD)			*p*-Value ^a^		
	Pre-COVID-19	Phase 1	Phase 2	Pre-COVID-19 vs. Phase 1	Pre-COVID-19 vs. Phase 2	Phase 1 vs. Phase 2
Esophageal cancer						
Total	8.82(2.75)	8.70(2.71)	7.67(3.65)	0.99	0.47	0.69
Stage 0	1.29(0.96)	0.90(0.74)	0.83(0.94)	0.46	0.30	0.98
Stage I	3.16(1.52)	3.10(1.45)	2.83(1.90)	0.99	0.81	0.92
Stage II	0.97(1.03)	0.80(0.79)	1.33(1.15)	0.88	0.54	0.45
Stage III	1.32(1.23)	1.50(1.27)	1.00(1.13)	0.91	0.72	0.61
Stage IV	2.08(1.60)	2.40(1.43)	1.67(1.44)	0.83	0.70	0.51
Medical Checkup cases ^b^	0.97(0.94)	1.10(0.88)	1.67(1.30)	0.93	0.11	0.40
Screening cases ^b^	3.39(1.57)	3.00(1.33)	2.42(1.62)	0.75	0.14	0.65
Symptomatic cases ^b^	3.84(1.84)	4.00(1.70)	3.50(2.07)	0.97	0.84	0.81

^a^ *p*-values were calculated using the Tukey–Kramer test (*p* < 0.05). ^b^ The detection process was categorized into medical checkups (conducted by the company, school, municipality, or at the patient’s own expense), screening (for patients who are at risk for cancers and who are being followed up for other diseases), and symptomatic cases.

**Table 7:** The average number of hepatocellular cancer patients per month per stage, the detection process, and comparison between each period.

	Number of Patients per Month, Mean (SD)			*p*-Value ^a^		
	Pre-COVID-19	Phase 1	Phase 2	Pre-COVID-19 vs. Phase 1	Pre-COVID-19 vs. Phase 2	Phase 1 vs. Phase 2
Hepatocellular carcinoma						
Total	8.89(3.17)	7.50(2.27)	6.67(2.61)	0.38	0.07	0.79
Stage I	4.87(2.28)	3.30(2.06)	3.58(1.78)	0.11	0.18	0.95
Stage II	1.84(1.72)	1.70(1.49)	1.58(1.08)	0.97	0.87	0.98
Stage III	1.34(1.12)	1.60(1.71)	0.83(0.72)	0.81	0.40	0.29
Stage IV	0.84(0.92)	0.90(1.10)	0.67(0.65)	0.98	0.83	0.82
Medical Checkup cases ^b^	0.55(0.60)	0.80(0.63)	0.58(0.67)	0.50	0.99	0.69
Screening cases ^b^	6.55(2.84)	4.60(1.65)	4.75(2.14)	0.09	0.09	0.99
Symptomatic cases ^b^	1.55(1.06)	1.70(1.16)	1.08(0.90)	0.92	0.37	0.36

^a^ *p*-values were calculated using the Tukey–Kramer test (*p* < 0.05). ^b^ The detection process was categorized into medical checkups (conducted by the company, school, municipality, or at the patient’s own expense), screening (for patients who are at risk for cancers and who are being followed up for other diseases), and symptomatic cases.

**Table 8:** The average number of biliary tract cancer patients per month per stage, the detection process, and comparison between each period.

	Number of Patients per Month, Mean (SD)			*p*-Value ^a^		
	Pre-COVID-19	Phase 1	Phase 2	Pre-COVID-19 vs. Phase 1	Pre-COVID-19 vs. Phase 2	Phase 1 vs. Phase 2
Biliary tract cancer						
Total	7.05(3.06)	6.20(2.25)	8.08(3.73)	0.72	0.58	0.34
Stage 0	0.34(0.58)	0.30(0.67)	0.17(0.58)	0.98	0.65	0.86
Stage I	1.29(1.41)	1.10(0.99)	0.75(0.87)	0.91	0.41	0.79
Stage II	1.84(1.48)	2.30(1.64)	2.25(1.22)	0.65	0.68	1.00
Stage III	1.55(1.37)	1.20(1.03)	2.58(1.24)	0.73	0.051	0.04 *
Stage IV	2.03(1.64)	1.30(1.16)	2.33(2.06)	0.44	0.84	0.32
Medical Checkup cases ^b^	0.45(0.69)	0.40(0.52)	0.42(0.90)	0.98	0.99	1.00
Screening cases ^b^	1.92(1.62)	1.30(1.16)	2.08(1.08)	0.47	0.94	0.43
Symptomatic cases ^b^	4.42(2.10)	4.40(1.84)	5.33(2.81)	1.00	0.43	0.59

^a^ *p*-values were calculated using the Tukey–Kramer test (* *p* < 0.05). ^b^ The detection process was categorized into medical checkups (conducted by the company, school, municipality, or at the patient’s own expense), screening (for patients who are at risk for cancers and who are being followed up for other diseases), and symptomatic cases.

## Data Availability

The data presented in this study are available on request from the corresponding author. Data are not publicly available because hospital-based cancer registries are not available to the public and can only be accessed on the hospital’s internal network.
